# MAP KINASE PHOSPHATASE1 promotes osmotolerance by suppressing PHYTOALEXIN DEFICIENT4-independent immunity

**DOI:** 10.1093/plphys/kiac131

**Published:** 2022-03-18

**Authors:** Kohei Uchida, Masahiro Yamaguchi, Kazuki Kanamori, Hirotaka Ariga, Kazuho Isono, Takuma Kajino, Keisuke Tanaka, Yusuke Saijo, Izumi Yotsui, Yoichi Sakata, Teruaki Taji

**Affiliations:** 1 Department of Bioscience, Tokyo University of Agriculture, Tokyo 156-8502, Japan; 2 Division of Plant Sciences, Institute of Agrobiological Science, NARO, Ibaraki 305-8602, Japan; 3 NODAI Genome Center, Tokyo University of Agriculture, Tokyo 156-8502, Japan; 4 Graduate School of Biological Sciences, Nara Institute of Science and Technology, Ikoma 630-0192, Japan

## Abstract

Initial exposure of plants to osmotic stress caused by drought, cold, or salinity leads to acclimation, termed acquired tolerance, to subsequent severe stresses. Acquired osmotolerance induced by salt stress is widespread across Arabidopsis (*Arabidopsis thaliana*) accessions and is conferred by disruption of a nucleotide-binding leucine-rich repeat gene, designated *ACQUIRED OSMOTOLERANCE*. De-repression of this gene under osmotic stress causes detrimental autoimmunity via *ENHANCED DISEASE SUSCEPTIBILITY1* and *PHYTOALEXIN DEFICIENT4* (*PAD4*). However, the mechanism underlying acquired osmotolerance remains poorly understood. Here, we isolated an acquired osmotolerance-defective mutant (*aod13*) by screening 30,000 seedlings of an ion beam-mutagenized M2 population of Bu-5, an accession with acquired osmotolerance. We found that *AOD13* encodes the dual-specificity phosphatase MAP KINASE PHOSPHATASE1 (MKP1), which negatively regulates MITOGEN-ACTIVATED PROTEIN KINASE3/6 (MPK3/6). Consistently, MPK3/6 activation was greater in *aod13* than in the Bu-5 wild-type (WT). The *aod13* mutant was sensitive to osmotic stress but tolerant to salt stress. Under osmotic stress, pathogenesis-related genes were strongly induced in *aod13* but not in the Bu-5 WT. Loss of *PAD4* in *pad4 aod13* plants did not restore acquired osmotolerance, implying that activation of immunity independent of PAD4 renders *aod13* sensitive to osmotic stress. These findings suggest that AOD13 (i.e. MKP1) promotes osmotolerance by suppressing the *PAD4*-independent immune response activated by MPK3/6.

## Teaser

Under osmotic stress, MAP KINASE PHOSPHATASE1 represses the MITOGEN-ACTIVATED PROTEIN KINASE3/6-dependent immune response that impairs osmotolerance of *Arabidopsis thaliana*.

## Introduction

Natural genetic variation in Arabidopsis (*Arabidopsis thaliana*) has facilitated the identification of genes governing complex traits such as growth, flowering, and stress tolerance in plants (Weigel, 2012). Wild populations of *A. thaliana* are distributed widely across the world (Alonso-Blanco and Koornneef, 2000). They provide diverse accessions useful for genetic studies, such as the 1001 Genomes collection of 1,135 accessions with whole-genome information (Consortium T 1001 G, 2016) and the RegMap collection of 1,307 accessions with 250K SNP information (Horton et al., 2012).

Osmotic stress caused by drought, salt, or cold decreases plant fitness. Acquired stress tolerance defines the ability of plants to withstand stress following an initial exposure to stress (Sung et al., 2003). We found previously that acquired osmotolerance after salt stress is widespread among *A. thaliana* accessions (Katori et al., 2010) and is conferred by a loss of *ACQUIRED OSMOTOLERANCE* (*ACQOS*; Ariga et al., 2017). Some accessions (e.g. Col-0) carrying functional *ACQOS* alleles are impaired in acquired osmotolerance, whereas other accessions (e.g. Bu-5) with nonfunctional *ACQOS* alleles show acquired osmotolerance. *ACQOS* is identical to *VARIATION IN COMPOUND TRIGGERED ROOT* growth response, which encodes a nucleotide-binding leucine-rich repeat (NLR) protein (Kim et al., 2012). In the absence of osmotic stress, *ACQOS* contributes to bacterial resistance, but under osmotic stress it causes detrimental autoimmunity via *ENHANCED DISEASE SUSCEPTIBILITY1* (*EDS1*) and *PHYTOALEXIN DEFICIENT4* (*PAD4*), thereby reducing osmotolerance (Ariga et al., 2017). Loss of *EDS1* or *PAD4* confers acquired osmotolerance in Col-0 plants (Ariga et al., 2017). Natural variations at the *ACQOS* locus suggest that a trade-off between biotic and abiotic stress adaptation maintains functional and nonfunctional *ACQOS* alleles (Ariga et al., 2017). However, the mechanism by which loss of *ACQOS* leads to acquired osmotolerance remains unclear.

Although osmotic tolerance often depends on the phytohormone abscisic acid (ABA) (Yoshida et al., 2014), the acquired osmotolerance of Bu-5 suppressed by ACQOS is ABA-independent (Ariga et al., 2017). *DEHYDRATION-REPONSIVE ELEMENT BINDING PROTEIN 1* (*DREB1*)/*C-REPEAT BINDING FACTOR* (*CBF*) and *DREB2* regulons include ABA-independent, osmotic stress-responsive genes (Yamaguchi-Shinozaki and Shinozaki, 1994; Stockinger et al., 1997; Maruyama et al., 2004; Nakashima et al., 2009). It has been widely accepted that *INDUCER OF CBP EXPRESSION1* (*ICE1*) mediates the induction of the *DREB*/*CBF* regulons, possibly through enhanced *DREB*/*CBF* expression (Chinnusamy, 2003; Ding et al., 2015). However, Kidokoro et al. (2020) reported that *DREB/CBF* repression in the *ice1-1* mutant is not dependent on the known ICE1 (R236H) mutation, but is achieved by DNA methylation-mediated gene silencing triggered by a T-DNA named *New ICE1* (*NICE1*). This highlights the need for re-examining the current ICE1–DREB/CBF regulatory model. *CALMODULIN BINDING TRANSCRIPTION ACTIVATOR 3* (*CAMTA3*) is involved in rapid induction of *CBF1* and *CBF2* (Doherty et al., 2009) and the DREB/CBF pathway is downregulated in the *camta1 camta2 camta3* (*camta123*) triple mutant under normal growth conditions (Kim et al., 2013). Involvement of ICE1–DREB/CBF regulation in acquired osmotolerance has yet to be tested.

Here, we ion-beam-mutagenized Bu-5 seeds and screened M2 population seedlings to identify acquired osmotolerance-defective (*aod*) mutants. We identified a gene contributing to acquired osmotolerance in *A. thaliana* and explored the mechanism by which this gene promotes osmotolerance.

## Results

### Isolation of *aod* mutants from Bu-5, an accession showing acquired osmotolerance

**Figure 1:**
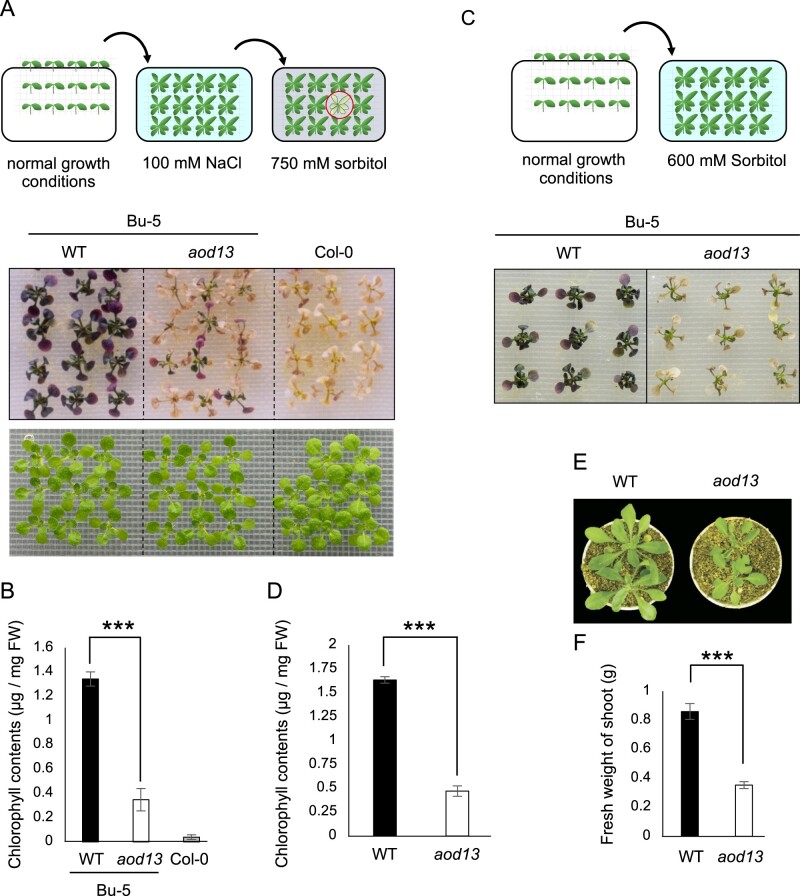
Identification of an acquired osmotolerance-defective mutant (*aod13*) of *A. thaliana*. A, Flow chart of the acquired osmotolerance assay (top). Salt-acclimated 2-week-old seedlings of accession Bu-5 were mesh-transferred to MS agar plates containing 750 mM sorbitol for 21 d. The seedlings showing osmo-hypersensitivity (red circle) were screened as *aod* mutants. Impaired acquired osmotolerance of the *aod13* mutant (bottom). WT Bu-5 is shown as an example of acquired osmotolerance and accession Col-0 is shown as an example of impaired acquired osmotolerance. Bottom panel: Two-week-old Bu-5 WT, *aod13*, and Col-0 WT seedlings under normal growth conditions. B, Chlorophyll contents of the seedlings shown in A. FW, fresh weight. C, Osmo-shock tolerance assay comparing *aod13* mutant and Bu-5 WT: 2-week-old seedlings were mesh-transferred to MS agar plates containing 600 mM sorbitol for 21 d. D, Chlorophyll contents of seedlings shown in (C). E, Examples of 4-week-old plants grown on soil under normal growth conditions. F, Fresh weight of plants grown as described in (E). Differences between WT (black bar) and *aod13* (white bar) were analyzed by Student’s *t* test (mean ± se, *n* = 8, ***P < 0.001).

When 7-d-old seedlings were pre-exposed to 100 mM NaCl for 7 d (acclimation period), *A. thaliana* accession Bu-5, but not Col-0, acquired osmotolerance to 750 mM sorbitol (Figure 1A), in agreement with our previous report (Ariga et al., 2017). We generated an ion-beam-mutagenized Bu-5 population and screened 30,000 M2 seedlings for *aod* mutants. We isolated several candidate mutants with the phenotype confirmed at the M3 generation. Acquired osmotolerance of *aod13* was significantly lower than that of Bu-5 wild-type (WT) (Figure 1, A and B). This mutant was also defective in osmo-shock tolerance assay (Figure 1, C and D). We observed no significant difference in ABA sensitivity between *aod13* and Bu-5 WT (Supplemental Figure S1). Overall, our data suggest that *AOD13* plays a positive role in osmotolerance via an ABA-independent pathway.

### Identification of the causal locus in *aod13*

**Figure 2:**
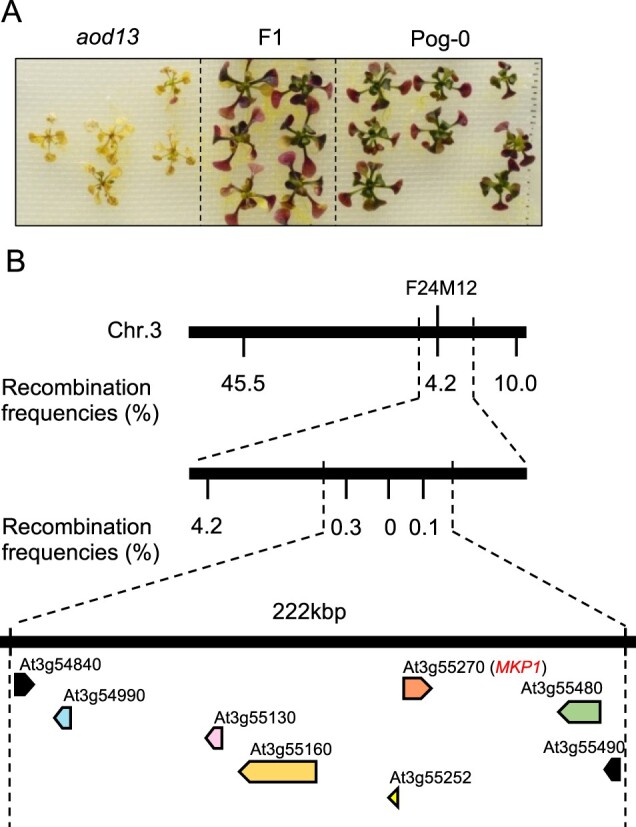
High-resolution mapping of the causal locus in *aod13*. A, Phenotypes of F_1_ progeny derived from a cross between *aod13* and accession Pog-0. B, High-resolution mapping of the causal locus in *aod13* by using F_2_ progeny between *aod13* and Pog-0. The scores indicate recombination frequencies (%). Genes located in the causal locus are shown as arrows. Arabidopsis Genome Initiative numbers are shown above the genes.

**Figure 3:**
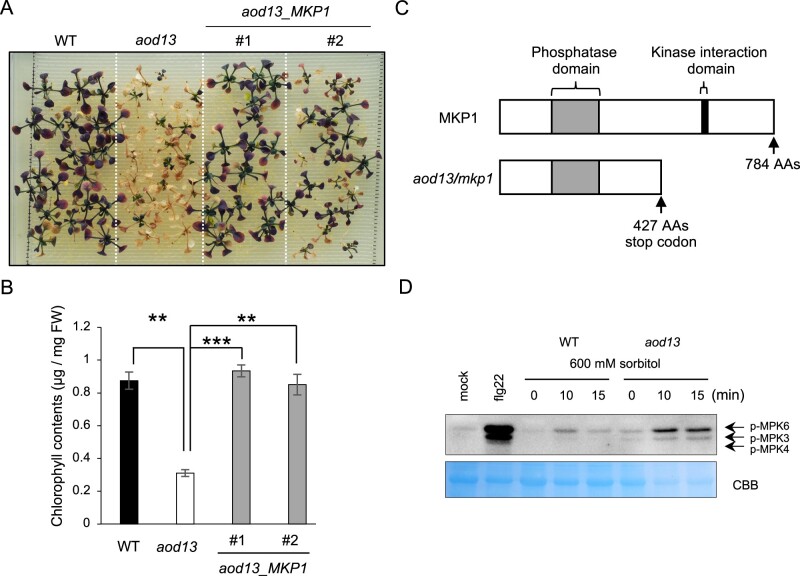
Identification of the causal gene in *aod13*. A, Complementation test performed by transforming *aod13* with *MKP1*. T_3_ homozygous plants transformed with *native promoter: MKP1* (*aod13*_*MKP1*) derived from Bu-5 WT were used in an acquired osmotolerance assay. B, Chlorophyll contents of WT, *aod13*, and *aod13*_*MKP1* as described in (A). Differences between Bu-5 WT and *aod13*, and between *aod13* and *aod13*_*MKP1* were analyzed by Student’s *t* test (mean ± se, *n* = 8, **P < 0.01, ***P < 0.001). C, Schematic representation of MKP1 and aod13 (i.e. mkp1) proteins. AAs, amino acids. D, Seven-day-old Bu-5 WT and *aod13* plants were treated with or without 600 mM sorbitol for the times indicated. As a positive control for activated MPK3/6 detection, the plants were treated with flg22, a conserved 22-amino-acid peptide derived from *P. syringae* flagellin, or water (mock). Proteins from treated leaf tissue were separated by SDS–PAGE and immunoblotting was performed using an anti-Phospho-p44/p42 MAPK (anti-pTEpY) (upper panel) to detect phosphorylated MAPKs. Extracted total proteins (input) stained with Coomassie brilliant blue served as a loading control (bottom panel).

We crossed *aod13* with accession Pog-0, which shows acquired osmotolerance and belongs to the same *ACQOS* haplogroup as Bu-5 (Ariga et al., 2017). The F_1_ progeny showed acquired osmotolerance like Pog-0, suggesting that the osmosensitive phenotype of *aod13* is recessive (Figure 2A). By high-resolution mapping, we found that the locus responsible for the osmosensitivity of *aod13* was located on the short arm of chromosome 3, near the simple sequence length polymorphism (SSLP) F24M12 within a 222-kbp region (Figure 2B). We revealed all sequence variations within this 222-kbp region of *aod13* by whole-genome sequencing of *aod13* and Bu-5 WT. We found nonsynonymous mutations in six genes within the region (Figure 2B). To identify the causal gene of *aod13*, we performed complementation tests in which each of the six genes derived from Bu-5, with their regulatory region, was introduced into *aod13*. Of them, only the introduction of *MKP1* (*aod13*_*MKP1*) restored acquired osmotolerance, like Bu-5 WT (Figure 3, A and B). We re-sequenced the *MKP1* coding region in *aod13* and found a 1-bp deletion, which caused a frameshift leading to truncation of the C-terminal binding domain for mitogen-activated protein kinases (MAPKs) (Figure 3C). These data suggest that *MKP1* was the causal gene for the osmosensitive phenotype of *aod13*.

### Dissecting MKP1 function under osmo-shock and salinity stress

MAPK signaling plays important roles in adaptive responses to a broad range of environmental stresses, such as salinity, UV-B radiation, cold, heavy metals, and pathogens (Colcombet and Hirt, 2008; Suarez Rodriguez et al., 2010; Besteiro et al., 2011). The magnitude and duration of MAPK activation are crucial in determining the physiological output of MAPK signaling. Specific dual-specificity phosphatases (MKPs) dephosphorylate conserved threonine and tyrosine residues in the activation loop of the MAPK kinase domain, thereby ensuring adequate intensity and duration of MAPK activation. In *A. thaliana*, MKP1 interacts with MAPKs (Ulm et al., 2002). MPK3/6 activities are significantly higher in *mkp1* than in Col-0 WT (Bartels et al., 2009). We assessed the activation status of MPK3/6 under osmo-shock stress and found that it was higher in *aod13* than in Bu-5 WT (Figure 3D).

**Figure 4:**
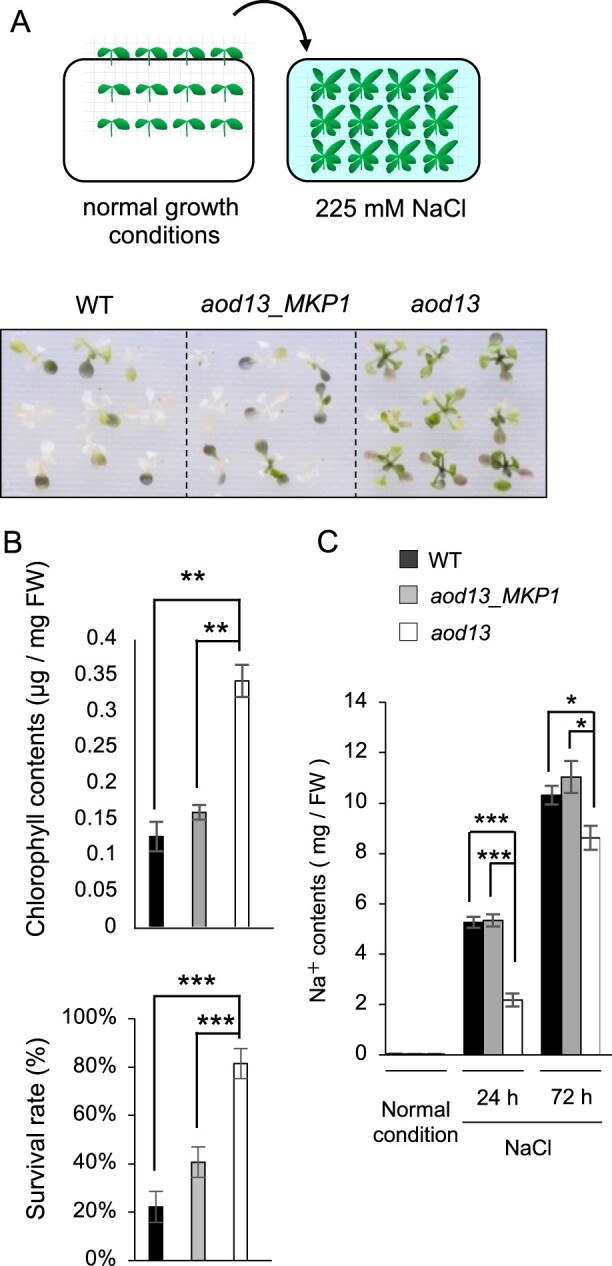
Salt tolerance of *aod13*. A, Flow chart of the salt-tolerance assay. Ten-day-old seedlings were mesh-transferred to MS agar plates containing 225 mM NaCl for 9 days (top). Salt tolerance of *aod13* was compared with Bu-5 WT and *aod13_MKP1* (bottom). B, Chlorophyll content (top) and survival rate (bottom) of WT, *aod13*_*MKP1*, and *aod13* treated as described in (A). C, Na^+^ contents in shoots under salt stress. Ten-day-old seedlings of WT, *aod13*_*MKP1*, and *aod13* were cultured on MS agar plates supplemented with 100 mM NaCl for 24 or 72 h. Differences between WT, *aod13*_*MKP1*, and *aod13* were analyzed by Student’s *t* test (mean ± se, *n* = 5, *P < 0.05, **P < 0.01, ***P < 0.001).

Ulm et al. (2002) reported enhanced tolerance to salt stress of *mkp1* mutants in the Ws background (Ulm et al., 2002). Salt sensitivity (as judged from chlorophyll content and survival rate) of *aod13* was lower than those of *aod13*_*MKP1* and Bu-5 WT (Figure 4, A and B and Supplemental Figure S2). Shoot Na^+^ contents under salt stress were lower in *aod13* than in Bu-5 WT or *aod13*_*MKP1* (Figure 4C), suggesting that suppression of Na^+^ accumulation in shoots contributed to the salt tolerance in *aod13*. Our analysis also showed that *aod13* was more tolerant than Bu-5 WT to salt stress, as indicated by the Na^+^ contents in the shoots.

### Effect of *DREB/CBF* repression in the acquired osmotolerance of *aod13*

**Figure 5:**
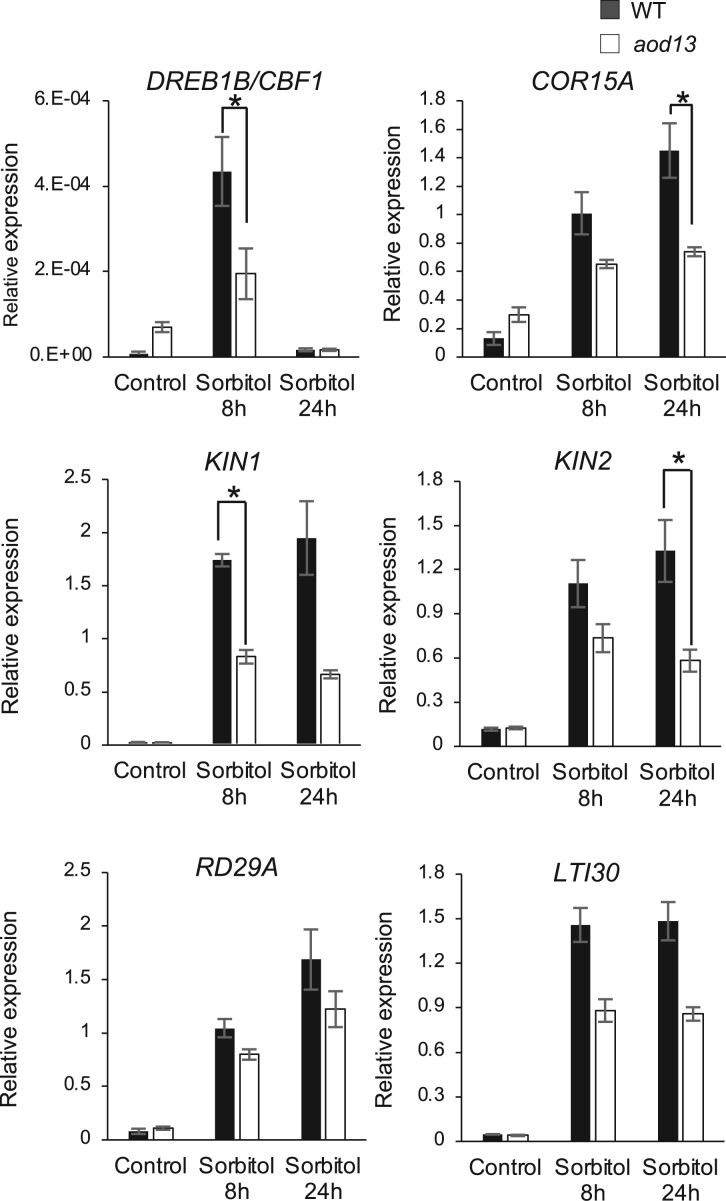
Expression profiles of the *DREB*/*CBF* regulon in *aod13*. Expression of *DREB1B*/*CBF1*, *COR15A*, *KIN1*, *KIN2*, *RD29A*, and *LYI30* in Bu-5 WT and *aod13* plants under normal (control) and acquired osmotolerance assay (100 mM NaCl for 7 d and subsequent 750 mM sorbitol) conditions; expression levels were determined by reverse transcription quantitative PCR relative to those of *Actin2* (mean ± se, *n* = 3). Differences between WT and *aod13* were analyzed by Student’s *t* test. *P < 0.05.

Recent studies reported that MPK3 and MPK6 phosphorylate ICE1, thereby inducing its degradation and reducing *DREB*/*CBF* expression and freezing tolerance in *A. thaliana* (Li et al., 2017; Zhao et al., 2017). These findings led us to hypothesize that loss of MKP1 function in *aod13* leads to hyper-activation of MPK3/6 and a decrease in *DREB/CBF* expression under osmotic stress, impairing the acquisition of osmotolerance. We analyzed the expression of a subset of the *DREB*/*CBF* regulon genes (*DREB1B*/*CBF1*, *COLD-REGULATED 15A* [*COR15A*], *STRESS-INDUCED PROTEIN* [*KIN1*, *KIN2*], *RESPONSIVE TO DESICCATION 29A* [*RD29A*], and *LOW-TEMPERATURE-RESPONSIVE PROTEIN 30*) under osmotic stress in *aod13* and Bu-5 WT and found that all these genes were induced, but the induction levels of *DREB1B*/*CBF1*, *COR15A*, *KIN1*, and *KIN2* were significantly lower in *aod13* than in Bu-5 WT (Figure 5).

**Figure 6:**
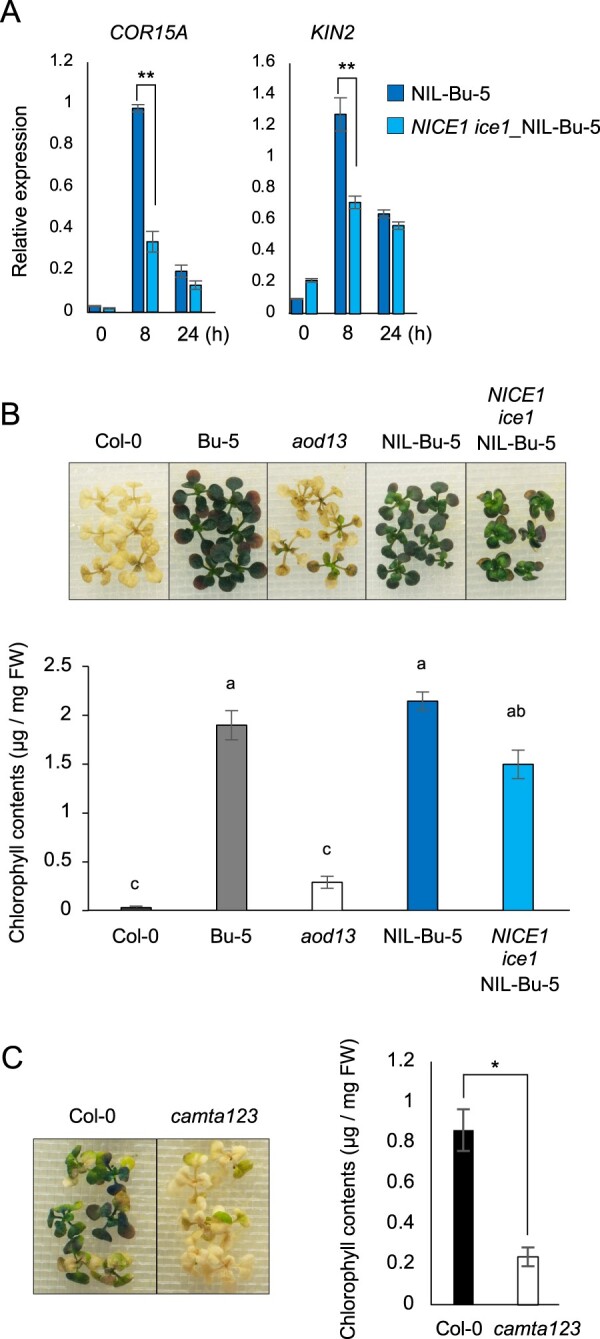
Contribution of the *DREB*/*CBF* regulon to the acquired osmotolerance. A, Expression levels of *COR15A* and *KIN2* in NIL-Bu-5 and *NICE1 ice1*_NIL-Bu-5 plants under normal and acquired osmotolerance assay (100 mM NaCl for 7 d and subsequent 750 mM sorbitol) conditions; expression levels were determined relative to those of *Actin2* by reverse transcription quantitative PCR. Differences between NIL-Bu-5 and *NICE1 ice1*_NIL-Bu-5 were analyzed by Student’s *t* test (mean ± se, *n* = 3, **P < 0.01). B, Assay of acquired osmotolerance of Col-0, Bu-5, *aod13*, NIL-Bu-5, and *NICE1 ice1*_NIL-Bu-5 (top panel). Chlorophyll contents of seedlings (bottom panel). Within each line, bars with different letters are signiﬁcantly different (*P* < 0.01, one-way ANOVA with post hoc Tukey’s HSD test, mean ± se, *n* = 3). C, Assay of acquired osmotolerance of Col-0-background *camta123* triple mutant (left panel). Chlorophyll contents of Col-0 and *camta123* triple mutants (right panel); Difference between Col-0 and *camta123* was analyzed by Student’s *t* test (mean ± se, *n* = 3, *P < 0.05).

To investigate whether the decrease in *DREB*/*CBF* induction impairs acquired osmotolerance in *aod13*, we crossed *ice1-1* with NIL-Bu-5, which carried the small chromosomal segment from Bu-5 containing the *ACQOS* region in the genetic background of Col-0 (Ariga et al., 2017), and thus introduced a *NICE1* transgene in the NIL-Bu-5 background. As expected, the induction of *COR15A* and *KIN2* was lower in *NICE1 ice1*_NIL-Bu-5 than in NIL-Bu-5 in acquired osmotolerance assay (Figure 6A). *NICE1 ice1*_NIL-Bu-5 was indistinguishable from NIL-Bu-5 in acquired osmotolerance assay, but its tolerance was significantly greater than that of *aod13* (Figure 6B). However, acquired osmotolerance was lower in the *camta123* triple mutant (Col-0), which exhibits strongly reduced expression of the *DREB*/*CBF* regulons (Kim et al., 2013; Zhao et al., 2016), than in Col-0 WT (Figure 6C). We inferred from these results that the *DREB/CBF* regulon plays a role in osmotolerance but not in *AOD13*-mediated osmotolerance.

#### *aod13* displays enhanced immune responses

**Figure 7:**
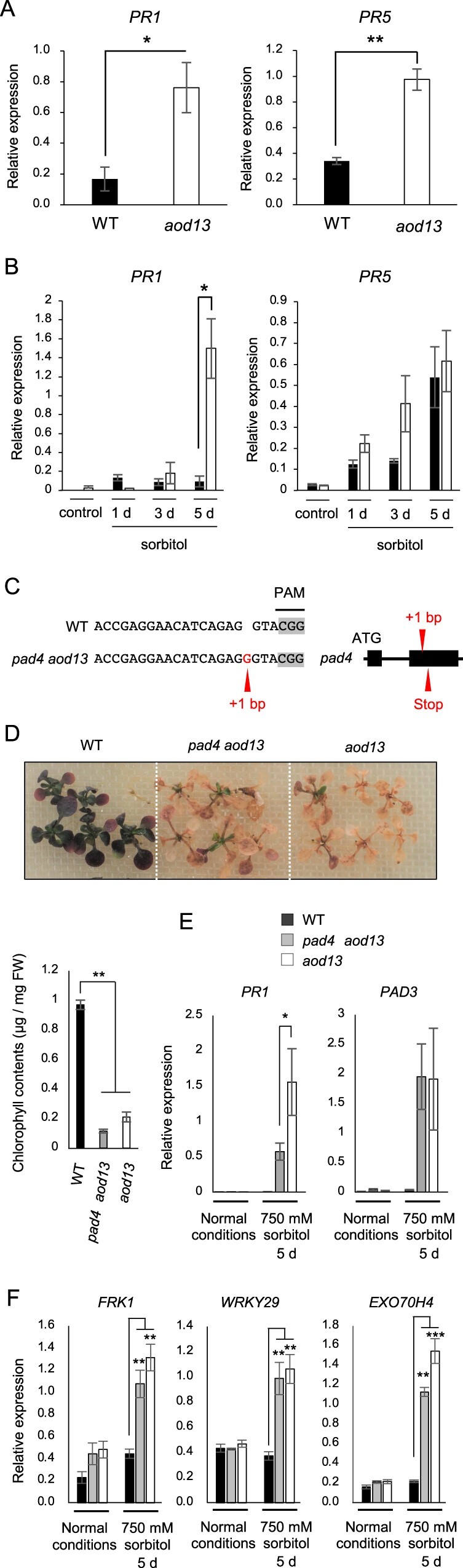
Impact of immune response on the acquired osmotolerance of *aod13*. A, Expression of *PR1* and *PR5* in Bu-5 WT and *aod13* plants grown on soil under normal conditions determined by RT-qPCR (mean ± se, *n* = 3). B, Expression of *PR1* and *PR5* in Bu-5 WT (black) and *aod13* (white) plants under normal (control) and acquired osmotolerance assay (100 mM NaCl for 7 d and subsequent 750 mM sorbitol) conditions; expression relative to *Actin2* was determined by RT-qPCR. C, Editing of the *PAD4* gene in *aod13* by the CRISPR/Cas9 system. *pad4 aod13* carries a 1-bp insertion mutation leading to a frameshift and premature termination codon. D, Acquired osmotolerance of WT, *pad4 aod13*, and *aod13* (top panel). Chlorophyll contents (bottom panel). E, Expression of *PR1* and *PAD3* in Bu-5 WT, *pad4 aod13*, and *aod13* plants under normal and acquired osmotolerance assay stress (100 mM NaCl for 7 d and subsequent 750 mM sorbitol) conditions; expression relative to *Actin2* was determined by RT-qPCR. F, Expression of *FRK1*, *WRKY29*, and *EXO70H4* in Bu-5 WT, *pad4 aod13*, and *aod13* plants under normal and acquired osmotolerance assay (100 mM NaCl for 7 d and subsequent 750 mM sorbitol) conditions; expression relative to *Actin2* was determined by RT-qPCR. Differences between Bu-5 WT and *aod13* or *pad4 aod13* were analyzed by Student’s *t* test (mean ± se, *n* = 3, *P < 0.05, **P < 0.01, ***P < 0.001).

Soil-grown *mkp1* plants display enhanced immune responses compared with Col-0 WT, including induction of salicylic acid (SA)-inducible defense-related genes, *PR1* and *PR5*, and enhanced resistance against the bacterial pathogen *Pseudomonas syringae* pv *tomato* DC3000 (Bartels et al., 2009). This is reminiscent of autoimmunity induced via *ACQOS* under osmotic stress, which likely impairs osmotolerance (Ariga et al., 2017). We determined the transcript levels of *PR1* and *PR5* in *aod13* plants. The transcript levels of both genes were higher in *aod13* than in Bu-5 WT in soil-gown plants without stress (Figure 7A). Osmotic stress induced *PR1* only in *aod13*, but *PR5* in both *aod13* and Bu-5 WT (Figure 7B). These findings imply defense activation in *aod13* exposed to osmotic stress, despite the lack of functional *ACQOS* in the Bu-5 background, as described previously in Col-0 WT carrying functional *ACQOS* (Ariga et al., 2017).

*ACQOS*-mediated autoimmunity is activated via *EDS1* and *PAD4*, and loss of *EDS1* or *PAD4* leads to acquired osmotolerance in Col-0 (Ariga et al., 2017). To test whether enhanced defense activation in *aod13* requires *PAD4* or not, we produced a *pad4 aod13* mutant by editing the *PAD4* gene in *aod13* with the CRISPR/Cas9 system (Supplemental Figure S3A; Hellens et al., 2002; Fauser et al., 2014; Yan et al., 2015; Endo et al., 2019). This mutant had a 1-bp insertion in the second exon of *PAD4*, leading to a frameshift and premature termination codon (Figure 7C and Supplemental Figure S3B). Unlike *pad4* (Col-0) (Ariga et al., 2017) and Bu-5 WT, *pad4 aod13* and *aod13* plants were not osmotolerant (Figure 7D). We then examined the transcript levels of *PR1* and *PAD3*; the latter encodes an enzyme for the biosynthesis of the antimicrobial camalexin and is regulated by an SA-independent MPK3/6 pathway (Qiu et al., 2008; Zhou et al., 2020). In acquired osmotolerance assay, *PR1* and *PAD3* were strongly induced in *pad4 aod13* and *aod3*, but not in Bu-5 WT, although *PR1* expression was lower in *pad4 aod13* than in *aod13* (Figure 7E). Sustained activation of MPK3 and MPK6 increases transcript levels of *FLG22-INDUCED RECEPTOR-LIKE KINASE1* (*FRK1*), *WRKY DNA-BINDING PROTEIN29* (*WRKY29*), and *EXOCYST SUBUNIT EXO70 FAMILY PROTEIN H4* (*EXO70H4*) in the absence of SA (Tsuda et al., 2013). To investigate whether MKP1 represses PAD4-independent immunity under osmotic stress, we assessed the transcript levels of *FRK1*, *WRKY29*, and *EXO70H4* in *aod13* and *pad4 aod13* plants. In acquired osmotolerance assay, all the transcript levels were higher in *aod13* and *pad4 aod13* than in WT plants under osmotic stress but not under normal conditions (Figure 7F). We concluded that under osmotic stress, the loss of *MKP1* function activates *PAD4*-independent defense responses and reduces osmotolerance in the *aod13* mutant.

## Discussion

Here, we describe the *aod13* mutant, in which loss of function of *MKP1* impairs acquired osmotolerance in the accession Bu-5 that lacks functional *ACQOS*. In *A. thaliana*, five dual-specificity MKPs, including MKP1, have been annotated (Bartels et al., 2010). *MKP* genes are often induced in response to stimuli triggering MAPK activation, thereby forming a negative feedback loop to dampen MAPK signaling (Martín et al., 2005). MKP1 can be phosphorylated by MPK6 (Park et al., 2011) and functions as a negative regulator of MPK3/6 (Bartels et al., 2009; Besteiro et al., 2011). Consistently, we show that a frameshift mutation in the *MKP1* gene in *aod13* increases MPK3/6 activation under osmotic stress. The *DREB*/*CBF* regulons constitute an important transcriptional regulatory network, which functions independently of ABA during various abiotic stress responses (Nakashima et al., 2009). MPK3/6 phosphorylate ICE1, which was initially thought to be a positive regulator of DREB/CBF, thereby leading to ICE1 degradation through the 26S proteasome pathway (Li et al., 2017; Zhao et al., 2017). Constitutive activation of MPK3/6 decreases the expression of the *DREB*/*CBF* regulons and freezing tolerance (Li et al., 2017; Zhao et al., 2017). Consistently, the expression levels of the tested *DREB*/*CBF* regulons were lower in *aod13* than in WT under osmotic stress. Given the contribution of *NICE1*, but not the *ice1* mutation per se, to *DREB*/*CBF* repression in *ice1-1* (Kidokoro et al., 2020), we also showed that the transcript levels of two *DREB*/*CBF* regulon genes in *NICE1 ice1*_NIL-Bu-5 were about half of those in NIL-Bu-5. Importantly, however, the acquired osmotolerance was not affected by *NICE1 ice1* in the NIL_Bu-5 background (Figure 6B). These findings point to the existence of a different mechanism from the *DREB*/*CBF* regulons, by which *AOD13* (i.e. *MKP1*) promotes acquired osmotolerance. The acquired osmotolerance of the *camta123* (Col-0) mutant is even lower than that of Col-0 WT, which is inherently defective in this respect. Future studies are required to test the combined effects of *camta123*, *ice1*, and *cbf123* mutations (Zhao et al., 2016) on the expression of *DREB/CBF* regulons and acquired osmotolerance in the NIL-Bu-5 background, to precisely assess the possible contribution of the *DREB*/*CBF* regulons to acquired osmotolerance.

Notably, CAMTA3 not only positively regulates *DREB*/*CBF* regulons, but also represses SA biosynthesis and SA-mediated immunity (Du et al., 2009). In *camta123*, SA accumulation is increased and *PR* genes are induced (Du et al., 2009; Kim et al., 2013). MPK3/6 directly phosphorylate CAMTA3, inducing its degradation (Jiang et al., 2020). Therefore, it is conceivable that CAMTA3 degradation via MPK3/6 activation contributes to the enhanced defense activation in *aod13* under osmotic stress (Figure 3D), in addition to the reduced expression of *DREB*/*CBF* regulons, and that both defects collectively cause the hypersensitivity to osmotic stress.

High concentrations of NaCl cause osmotic stress and also Na^+^ toxicity. Enhanced tolerance to salt and decreased tolerance to osmotic stress in *aod13* suggest that MKP1 is a negative regulator for salt stress and positive regulator for osmotic stress. MPK6 physically interacts with and phosphorylates the plasma membrane Na^+^/H^+^ antiporter SALT OVERLY SENSITIVE1 (SOS1), a master regulator of salt tolerance (Yu et al., 2010), which suppresses Na^+^ accumulation in shoots (Ji et al., 2013). The salt tolerance of *mkp1* may be attributable to the activation of SOS1 via elevation of MPK6 activity. Interestingly, following pre-exposure to 100 mM NaCl, *aod13* acquired tolerance to high NaCl concentrations (300–400 mM; equivalent to 750 mM sorbitol in terms of osmotic stress) which severely damaged WT and *aod13_MKP1* plants (Supplemental Figure S2). This finding supports MKP1 as a negative regulator of salinity stress tolerance, whether the plants are exposed directly or after acclimation.

The forward genetic screening of *aod* mutants identified *AOD13/MKP1* as playing important roles in osmotolerance of *A.* *thaliana* by suppressing PAD4-independent immunity activated by MKP3/6 under osmotic stress.

## Materials and methods

### Plant materials and growth conditions

Arabidopsis (*A.* *thaliana*) seeds (Bu-5 or Col-0) were sown on agar (0.8%, w/v) plates containing full-strength Murashige and Skoog (MS) salts with a vitamin mixture (10 mg L^−1^ myoinositol, 200 μg L^−1^ glycine, 50 μg L^−1^ nicotinic acid, 50 μg L^−1^ pyridoxine hydrochloride, and 10 μg L^−1^ thiamine hydrochloride, pH 5.7) and 1% (w/v) sucrose. Plates were sealed with surgical tape; the seeds were stratified at 4°C for 4–7 d and then transferred to a growth chamber (80 μmol photons m^2^ s^−1^; 16-h/8-h light/dark cycle; 22°C) for germination and growth.

Seeds of the following *A. thaliana* mutants were obtained from the Arabidopsis Biological Resource Center (Ohio State University): *ice-1-1* (CS67843); *dreb1b/cbf1* (CS866868); and *camta123* (CS71606).

### Irradiation

Bu-5 seeds were irradiated in the azimuthally varying field cyclotron at the Japan Atomic Energy Agency (Takasaki, Japan). To select the appropriate dose, we irradiated the seeds with carbon ion beams in a dose range of 25–250 Gy and assessed plant development. Doses of 200 Gy or higher inhibited secondary leaf development or induced sterility. Thus, we irradiated seeds at 150 Gy in a single layer within a plastic bag.

### Stress treatment for the acquired osmotolerance assay

Seven-day-old seedlings grown on nylon mesh (990 μm) on an MS agar plate were mesh-transferred to a plate supplemented with 100 mM NaCl for 7 d. The seedlings were then mesh transferred to a plate supplemented with 750 mM sorbitol for 14 d.

### Stress treatments for salt tolerance and osmo-shock tolerance assays

Ten-day-old seedlings grown on nylon mesh (990 μm) on an MS agar plate were mesh-transferred to a plate supplemented with 225 mM NaCl for 7 d for the salt-tolerance assay or mesh-transferred to a plate supplemented with 600 mM sorbitol for 21 d for the osmo-shock tolerance assay.

### Chlorophyll determination

Aerial parts of five randomly chosen seedlings from each group treated with or without various stresses were harvested and then homogenized in cold acetone. Chlorophyll content was determined using the method described by Porra et al. (1989). Differences were analyzed by Student’s *t* test.

### Genetic mapping of *aod13*

The mutant *aod13* was crossed with an osmo-tolerant accession, Pog-0, and the resulting F_1_ progeny were selfed to generate F_2_ populations. Genomic DNA was prepared from individual F_2_ plants with the recessive phenotype for use as PCR templates. The SSLP markers listed in Supplemental Table S1 were used for mapping. PCR conditions were as follows: (94°C for 2 min) 1 cycle, (94°C for 20 s, 52°C–55°C for 20 s, 72°C for 20 s) 34 cycles, and (72°C for 2 min) 1 cycle. The microsatellites were fractionated in a 5%–7% (w/v) agarose gel and the recombination frequencies (%) were calculated from the band pattern.

### DNA library construction and sequencing of *aod13* and Bu-5

Each genomic DNA (1 μg) was sheared to an average fragment size of 300 bp by using an Adaptive Focused Acoustics sonicator (Covaris, Inc., Woburn, MA, USA). After purification with a QIAquick PCR Purification Kit (Qiagen, Valencia, CA, USA), DNA libraries were constructed using a NEBNext DNA Library Prep Master Mix Set for Illumina and NEBNext Multiplex Oligos for Illumina (New England Biolabs, Ipswich, MA, USA). Briefly, the fragmented DNAs were end-repaired, dA-tailed, and ligated with the NEBNext adapter. Size selection was conducted using AMPure XP magnetic beads (Beckman Coulter, Brea, CA, USA) following the manufacturer’s instructions. The adapter-ligated DNAs were amplified by eight cycles of high-fidelity PCR with different index primers. The PCR products were cleaned up using the AMPure XP magnetic beads. Each library’s quality and concentration were assessed using an Agilent Bioanalyzer 2100 (Agilent Technologies, Santa Clara, CA, USA). The concentrations were then determined more precisely by real-time quantitative PCR (qPCR) using a KAPA Library Quantification Kit (Kapa Biosystems, Wilmington, MA, USA).

All libraries were diluted to 10 nM and then mixed in equal amounts. The library mixture was sequenced by 1 × 100-bp single-read sequencing using an Illumina HiSeq 2500 sequencing system (Illumina, San Diego, CA, USA). Reads in FASTQ format were generated using bcl2fastq2 Conversion Software version 2.18 (Illumina). The read data were submitted to the DNA Data Bank of Japan (DDBJ) Read Archive (Accession Number: DRA012094).

### Detection of nonsynonymous mutations

Adapter sequences in each read were removed using CLC Genomics Workbench version 9.0 (Qiagen, Hilden, Germany). Then, the clean read data were mapped to the reference genome of *A. thaliana* (TAIR10) retrieved from the Ensembl Plants database (https://plants.ensembl.org/index.html). Mapping parameters were as follows: (1) mismatch cost, 2; (2) insertion cost, 3; (3) deletion cost, 3; (4) length fraction, 0.9; and (5) similarity fraction, 0.9. After local realignment of the mapped reads, PCR duplicate reads were discarded. These processes were carried out with default parameters. Variant calling was performed using the built-in tool, “Basic Variant Detection,” which detects single-nucleotide polymorphisms and insertion–deletion mutations. The detection parameters were as follows: (1) minimum coverage, 15; (2) minimum count, 2; and (3) minimum frequency, 50%. Nonsynonymous mutations between the two lines, Bu-5 and *aod13*, were detected by the built-in tools, “Amino Acid Changes” and “Annotate with Overlap Information.”

### Plasmid construction and transformation

For complementation analysis, the genomic region of *MKP1* (2.0-kb upstream of the ATG initiation codon and 1.0-kb downstream of the termination codon of Bu-5) was amplified by PCR with XbaI linker primers and cloned into the XbaI sites introduced into the binary vector pBIG2113 with 35S promoter deleted by HindIII and EcoRI digestion. The construct was introduced into *Agrobacterium tumefaciens* strain GV3101. Plants were transformed with the agrobacteria using the floral dip method. Primers for cloning are listed in Supplemental Table S2. Transgenic plants were selected on MS agar plates containing 200 µg mL^−1^ claforan and 20 µg mL^−1^ hygromycin. Ten-day-old seedlings (T_1_ plants) were transferred to soil pots.

### MAP kinase assay

One-week-old *A. thaliana* seedlings were removed from plates and placed in the other plates filled with or without 600 mM sorbitol solution and then proteins were extracted using extraction buffer (50 mM Tris–HCl [pH 7.5], 10 mM MgCl_2_, 15 mM EGTA, 100 mM NaCl, 2 mM dithiothreitol, 1 mM sodium fluoride, 0.5 mM Na_3_VO_4_, 30 mM β-glycerophosphate, 0.1% [v/v] NP-40 detergent, and one Complete tablet, EDTA-free per 50 mL) (Roche, Basel, Switzerland). Total proteins (30 μg) were separated by electrophoresis on 10% (v/v) acrylamide (Wako, Tokyo, Japan) and transferred to Immobilon-FL PVDF membrane (Merck, Darmstadt, Germany) and the blotted membrane was stained with Coomassie Brilliant Blue to verify equal loading. Phosphorylated MAPK proteins were detected by immunoblot analysis with antiphospho-p44/42 MAPK (Erk1/2) (Thr202/Tyr204) (D13.14.4E) rabbit mAb (Cell Signaling Technology, MA, USA). Blotting Grade Anti-Rabbit IgG (H + L) (Human IgG Absorbed) Horseradish Peroxidase Conjugate (Bio-Rad, Hercules, CA, USA) was used as secondary antibody.

### Measurement of Na^+^ contents

Seven-day-old Bu-5 and *aod13* seedlings grown on nylon mesh on MS agar plates were mesh transferred to plates supplemented with 100 mM NaCl. Aerial parts of the seedlings were harvested at 0, 1, and 3 d after the transfer. Plants were soaked in 5 mL of sterile distilled water for 5 s. The solution was then boiled for 15 min, passed through a 0.2-μm filter (Toyo Roshi, Tokyo, Japan), and diluted 20 times with distilled water. The Na^+^ content of the solution was measured by using a Dionex IonPac CS12A column on a Dionex ICS-900 ion chromatography system (Dionex, Sunnyvale, CA, USA). Differences were analyzed by Student’s *t* test.

### RT-qPCR

Total RNA (2 µg) was isolated with a RNeasy Plant Mini Kit (Qiagen, Hilden, Germany), treated with DNase I (Invitrogen, Waltham, MA, USA), and used as a template to synthesize first-strand cDNA using SuperScript II Reverse Transcriptase (Invitrogen) and an oligo dT primer. Then, reverse transcription-quantitative PCR (RT-qPCR) was performed using a LightCycler 96 (Roche Diagnostics) with FastStart Essential DNA Green Master (Roche Diagnostics) in a total volume of 12 µL under the following conditions: 95°C for 10 min followed by 45–50 cycles of 95°C for 20 s, 54°C for 20 s, and 72°C for 20 s. *Actin2* was used as an internal standard. Primers and their efficiencies are listed in Supplemental Table S3. Differences were analyzed by Student’s *t* test.

### Generation of the *pad4 aod13* mutant using the CRISPR-Cas9 system

For the construction of all-in-one CRISPR-Cas9 vectors, we generated pGK-YC9 (accession number: LC636137, see also Supplemental Figure S3A). For sgRNA expression, a complementary oligonucleotide pair comprising the target sequence of the *PAD4* gene (ACCGAGGAACATCAGAGGTA) was annealed and then inserted into the *Bsa*I site between the *AtU6-26* promoter and the sgRNA scaffold in pGK-YC9. To isolate *aod13* plants carrying a mutation in the *PAD4* gene from among T1 seedlings, T7 endonuclease 1 (T7E1, New England Biolabs) assay was performed using primers (F: GACCTCGTTCCTAGAAGCAGCAA, R: CCTAGCTGCTCTTCTGATGCAT). Null segregants (lacking T-DNA) carrying a homozygous *PAD4* mutation were detected in the T2 generation by PCR and confirmed by Sanger sequencing. The T3 generation of *pad4 aod13* plants was used in this study.

## Accession numbers

The read data were submitted to the DDBJ Read Archive (accession number DRA012094). Sequence data of the *MKP1* gene can be found in the GenBank/EMBL data libraries under accession number At3g55270. Accession numbers for genes used in RT-qPCR experiments are listed in Supplemental Table S3.

## Supplemental data

The following materials are available in the online version of this article.

**Supplemental Figure S1.** ABA sensitivity of *aod13*.

**Supplemental Figure S2.** Tolerance of *aod13* to severe salt stress.

**Supplemental Figure S3.** Editing of the *PAD4* gene in *aod13* using the CRISPR/Cas9 system.

**Supplemental Table S1.** Primer sets for the SSLP markers used in this study.

**Supplemental Table S2.** Primers for cloning and sequencing of *MKP1.*

**Supplemental Table S3.** Primer sets for RT-qPCR.

## Supplementary Material

kiac131_Supplementary_Data

## Notes

These authors contributed equally (K.U. and M.Y.).

K.U. and T.T. initiated, conceived, and coordinated the project. K.U. isolated the aod13 mutant and identified the causal gene. K.U., M.Y., K.I., T.K., and K.K. performed the physiological and biochemical analyses. H.A. performed the gene editing by the CRISPR/Cas9 system. K.T. performed the genome sequencing. T.T. wrote the manuscript with assistance from I.Y.,Y. Saijo, and Y. Sakata.

The author responsible for distribution of materials integral to the findings presented in this article in accordance with the policy described in the Instructions for Authors (https://academic.oup.com/plphys/pages/general-instructions) is: Teruaki Taji (t3teruak@nodai.ac.jp).

## References

[kiac131-B1] Alonso-Blanco C , Koornneef M (2000) Naturally occurring variation in Arabidopsis: an underexploited resource for plant genetics. Trend Plant Sci 5**:** 22–29 10.1016/s1360-1385(99)01510-1 10637658

[kiac131-B2] Ariga H , Katori T, Tsuchimatsu T, Hirase T, Tajima Y, Parker JE, Alcázar R, Koornneef M, Hoekenga O, Lipka AE, et al (2017) NLR locus-mediated trade-off between abiotic and biotic stress adaptation in Arabidopsis. Nat Plants 3: 17072 28548656 10.1038/nplants.2017.72

[kiac131-B3] Bartels S , Anderson JC, Besteiro MAG, Carreri A, Hirt H, Buchala A, Métraux JP, Peck SC, Ulm R (2009) MAP kinase phosphatase1 and protein tyrosine phosphatase1 are repressors of salicylic acid synthesis and SNC1-mediated responses in Arabidopsis. Plant Cell 21**:** 2884–2897 19789277 10.1105/tpc.109.067678 PMC2768924

[kiac131-B4] Bartels S , Besteiro MAG, Lang D, Ulm R (2010) Emerging functions for plant MAP kinase phosphatases. Trend Plant Sci 15**:** 322–329 10.1016/j.tplants.2010.04.003 20452268

[kiac131-B5] Besteiro MAG , Bartels S, Albert A, Ulm R (2011) Arabidopsis MAP kinase phosphatase 1 and its target MAP kinases 3 and 6 antagonistically determine UV-B stress tolerance, independent of the UVR8 photoreceptor pathway. Plant J 68**:** 727–737 21790814 10.1111/j.1365-313X.2011.04725.x

[kiac131-B7] Chinnusamy V (2003) ICE1: a regulator of cold-induced transcriptome and freezing tolerance in Arabidopsis. Genes Dev 17**:** 1043–1054 12672693 10.1101/gad.1077503 PMC196034

[kiac131-B8] Colcombet J , Hirt H (2008) Arabidopsis MAPKs: a complex signalling network involved in multiple biological processes. Biochem J 413: 217–226 18570633 10.1042/BJ20080625

[kiac131-B9] Consortium T 1001 G (2016) 1,135 Genomes reveal the global pattern of polymorphism in *Arabidopsis thaliana*. Cell 166: 481–491 27293186 10.1016/j.cell.2016.05.063 PMC4949382

[kiac131-B10] Ding Y , Li H, Zhang X, Xie Q, Gong Z, Yang S (2015) OST1 kinase modulates freezing tolerance by enhancing ICE1 stability in Arabidopsis. Dev Cell 32: 278–289 25669882 10.1016/j.devcel.2014.12.023

[kiac131-B11] Doherty CJ , Buskirk HAV, Myers SJ, Thomashow MF (2009) Roles for Arabidopsis CAMTA transcription factors in cold-regulated gene expression and freezing tolerance. Plant Cell 21**:** 972–984 19270186 10.1105/tpc.108.063958 PMC2671710

[kiac131-B12] Du L , Ali GS, Simons KA, Hou J, Yang T, Reddy ASN, Poovaiah BW (2009) Ca^2+^/calmodulin regulates salicylic-acid-mediated plant immunity. Nature 457**:** 1–6 10.1038/nature07612 19122675

[kiac131-B13] Endo M , Mikami M, Endo A, Kaya H, Itoh T, Nishimasu H, Nureki O, Toki S (2019) Genome editing in plants by engineered CRISPR–Cas9 recognizing NG PAM. Nat Plants 5**:** 14–17 30531939 10.1038/s41477-018-0321-8

[kiac131-B14] Fauser F , Schiml S, Puchta H (2014) Both CRISPR/Cas‐based nucleases and nickases can be used efficiently for genome engineering in Arabidopsis thaliana. Plant J 79**:** 348–359 24836556 10.1111/tpj.12554

[kiac131-B15] Hellens RP , Edwards EA, Leyland NR, Bean S, Mullineaux PM (2002) pGreen: a versatile and flexible binary Ti vector for Agrobacterium-mediated plant transformation. Plant Mol Biol 819**:** 832 10.1023/a:1006496308160 10890530

[kiac131-B16] Horton MW , Hancock AM, Huang YS, Toomajian C, Atwell S, Auton A, Muliyati NW, Platt A, Sperone FG, Vilhjálmsson BJ, et al (2012) Genome-wide patterns of genetic variation in worldwide *Arabidopsis thaliana* accessions from the RegMap panel. Nat Genet 44**:** 212–216 22231484 10.1038/ng.1042 PMC3267885

[kiac131-B17] Ji H , Pardo JM, Batelli G, Oosten MJV, Bressan RA, Li X (2013) The Salt Overly Sensitive (SOS) pathway: established and emerging roles. Mol Plant 6**:** 275–286 23355543 10.1093/mp/sst017

[kiac131-B18] Jiang X , Hoehenwarter W, Scheel D, Lee J (2020) Phosphorylation of the CAMTA3 transcription factor triggers its destabilization and nuclear export. Plant Physiol 184**:** 00795.2020 10.1104/pp.20.00795 PMC7536672 32769161

[kiac131-B19] Katori T , Ikeda A, Iuchi S, Kobayashi M, Shinozaki K, Maehashi K, Sakata Y, Tanaka S, Taji T (2010) Dissecting the genetic control of natural variation in salt tolerance of *Arabidopsis thaliana* accessions. J Exp Bot 61**:** 1125–1138 20080827 10.1093/jxb/erp376 PMC2826654

[kiac131-B20] Kidokoro S , Kim JS, Ishikawa T, Suzuki T, Shinozaki K, Yamaguchi-Shinozaki K (2020) DREB1A/CBF3Is repressed by transgene-induced DNA methylation in the Arabidopsis ice1-1 mutant. Plant Cell 32**:** 1035–1048 32034036 10.1105/tpc.19.00532 PMC7145508

[kiac131-B21] Kim TH , Kunz HH, Bhattacharjee S, Hauser F, Park J, Engineer C, Liu A, Ha T, Parker JE, Gassmann W, et al (2012) Natural variation in small molecule-induced TIR-NB-LRR signaling induces root growth arrest via EDS1- and PAD4-complexed R protein VICTR in Arabidopsis. Plant Cell 24**:** 5177–5192 23275581 10.1105/tpc.112.107235 PMC3556982

[kiac131-B22] Kim Y , Park S, Gilmour SJ, Thomashow MF (2013) Roles of CAMTA transcription factors and salicylic acid in configuring the low‐temperature transcriptome and freezing tolerance of Arabidopsis. Plant J 75**:** 364–376 23581962 10.1111/tpj.12205

[kiac131-B23] Li H , Ding Y, Shi Y, Zhang X, Zhang S, Gong Z, Yang S (2017) MPK3- and MPK6-mediated ICE1 phosphorylation negatively regulates ICE1 stability and freezing tolerance in Arabidopsis. Dev Cell 43**:** 630–642.e4 29056553 10.1016/j.devcel.2017.09.025

[kiac131-B24] Martín H , Flández M, Nombela C, Molina M (2005) Protein phosphatases in MAPK signalling: we keep learning from yeast. Mol Microbiol 58**:** 6–16 16164545 10.1111/j.1365-2958.2005.04822.x

[kiac131-B25] Maruyama K , Sakuma Y, Kasuga M, Ito Y, Seki M, Goda H, Shimada Y, Yoshida S, Shinozaki K, Yamaguchi-Shinozaki K (2004) Identification of cold-inducible downstream genes of the Arabidopsis DREB1A/CBF3 transcriptional factor using two microarray systems. Plant J 38**:** 982–993 15165189 10.1111/j.1365-313X.2004.02100.x

[kiac131-B26] Nakashima K , Ito Y, Yamaguchi-Shinozaki K (2009) Transcriptional regulatory networks in response to abiotic stresses in Arabidopsis and grasses. Plant Physiol 149**:** 88–95 19126699 10.1104/pp.108.129791 PMC2613698

[kiac131-B27] Park HC , Song EH, Nguyen XC, Lee K, Kim KE, Kim HS, Lee SM, Kim SH, Bae DW, Yun DJ, et al (2011) Arabidopsis MAP kinase phosphatase 1 is phosphorylated and activated by its substrate AtMPK6. Plant Cell Rep 30**:** 1523–1531 21455789 10.1007/s00299-011-1064-4

[kiac131-B28] Porra RJ , Thompson WA, Kriedemann PE (1989) Determination of accurate extinction coefficients and simultaneous equations for assaying chlorophylls a and b extracted with four different solvents: verification of the concentration of chlorophyll standards by atomic absorption spectroscopy. Biochim Biophys Acta 975**:** 384–394

[kiac131-B29] Qiu J , Fiil BK, Petersen K, Nielsen HB, Botanga CJ, Thorgrimsen S, Palma K, Suarez‐Rodriguez MC, Sandbech‐Clausen S, Lichota J, et al (2008) Arabidopsis MAP kinase 4 regulates gene expression through transcription factor release in the nucleus. EMBO J 27**:** 2214–2221 18650934 10.1038/emboj.2008.147 PMC2519101

[kiac131-B30] Stockinger EJ , Gilmour SJ, Thomashow MF (1997) *Arabidopsis thaliana* CBF1 encodes an AP2 domain-containing transcriptional activator that binds to the C-repeat/DRE, a cis-acting DNA regulatory element that stimulates transcription in response to low temperature and water deficit. Proc Natl Acad Sci USA 94**:** 1035–1040 9023378 10.1073/pnas.94.3.1035 PMC19635

[kiac131-B31] Suarez Rodriguez MC , Petersen M, Mundy J (2010) Mitogen-activated protein kinase signaling in plants. Ann Rev Plant Biol 61**:** 621–649 20441529 10.1146/annurev-arplant-042809-112252

[kiac131-B32] Sung DY , Kaplan F, Lee KJ, Guy CL (2003) Acquired tolerance to temperature extremes. Trend Plant Sci 8**:** 179–187 10.1016/S1360-1385(03)00047-5 12711230

[kiac131-B33] Tsuda K , Mine A, Bethke G, Igarashi D, Botanga CJ (2013) Dual regulation of gene expression mediated by extended MAPK activation and salicylic acid contributes to robust innate immunity in *Arabidopsis thaliana*. PLoS Genet 9**:** e1004015 24348271 10.1371/journal.pgen.1004015 PMC3861249

[kiac131-B34] Ulm R , Ichimura K, Mizoguchi T, Peck SC, Zhu T, Wang X, Shinozaki K, Paszkowski J (2002) Distinct regulation of salinity and genotoxic stress responses by Arabidopsis MAP kinase phosphatase 1. EMBO J 21**:** 6483–6493 12456655 10.1093/emboj/cdf646 PMC136950

[kiac131-B35] Weigel D (2012) Natural variation in Arabidopsis: from molecular genetics to ecological genomics. Plant Physiol 158**:** 2–22 22147517 10.1104/pp.111.189845 PMC3252104

[kiac131-B36] Yamaguchi-Shinozaki K , Shinozaki K (1994) A novel cis-acting element in an Arabidopsis gene is involved in responsiveness to drought, low-temperature, or high-salt stress. Plant Cell 6**:** 251–264 8148648 10.1105/tpc.6.2.251 PMC160431

[kiac131-B37] Yan L , Wei S, Wu Y, Hu R, Li H, Yang W, Xie Q (2015) High-efficiency genome editing in Arabidopsis using YAO promoter-driven CRISPR/Cas9 system. Mol Plant 8**:** 1820–1823 26524930 10.1016/j.molp.2015.10.004

[kiac131-B38] Yoshida T , Mogami J, Yamaguchi-Shinozaki K (2014) ABA-dependent and ABA-independent signaling in response to osmotic stress in plants. Curr Opin Plant Biol 21**:** 133–139 25104049 10.1016/j.pbi.2014.07.009

[kiac131-B39] Yu L , Nie J, Cao C, Jin Y, Yan M, Wang F, Liu J, Xiao Y, Liang Y, Zhang W (2010) Phosphatidic acid mediates salt stress response by regulation of MPK6 in *Arabidopsis thaliana*. New Phytol 188**:** 762–773 20796215 10.1111/j.1469-8137.2010.03422.x

[kiac131-B40] Zhao C , Wang P, Si T, Hsu CC, Wang L, Zayed O, Yu Z, Zhu Y, Dong J, Tao WA, et al (2017) MAP kinase cascades regulate the cold response by modulating ICE1 protein stability. Dev Cell 43**:** 618–629.e5 29056551 10.1016/j.devcel.2017.09.024 PMC5716877

[kiac131-B41] Zhao C , Zhang Z, Xie S, Si T, Li Y, Zhu JK (2016) Mutational evidence for the critical role of CBF transcription factors in cold acclimation in Arabidopsis. Plant Physiol 171**:** 2744–2759 27252305 10.1104/pp.16.00533 PMC4972280

[kiac131-B42] Zhou J , Wang X, He Y, Sang T, Wang P, Dai S, Zhang S, Meng X (2020) Differential phosphorylation of the transcription factor WRKY33 by the protein kinases CPK5/CPK6 and MPK3/MPK6 cooperatively regulates camalexin biosynthesis in Arabidopsis. Plant Cell 32**:** 2621–2638 32439826 10.1105/tpc.19.00971 PMC7401014

