# Supplementary material for: MAP KINASE PHOSPHATASE1 promotes osmotolerance by suppressing PHYTOALEXIN DEFICIENT4-independent immunity
Source: Plant Physiol. 2022 Mar 18;189(2):1128–38. doi: 10.1093/plphys/kiac131 (PMC9157078; doi:10.1093/plphys/kiac131)
Supplement: kiac131_Supplementary_Data [file kiac131_supplementary_data.zip › Supplemental Tables.pdf]

Supplemental Table S1. SSLP markers used in this study

Number of chromosome	Marker name	Sequence (5' → 3')
chr.1	JV26/27 F	CAAGAGATTGCAACATCCACA
	JV26/27 R	AAGCTCCTTGGATCCGATTT
	SO392 F	GTTGATCGCAGCTTGATAAGC
	SO392 R	TTTGGAGTTAGACACGGATCTG
	CIW1 F	ACATTTTCTCAATCCTTACTC
	CIW1 R	GAGAGCTTCTTTATTTGTGAT
	NGA111 F	TGTTTTTTAGGACAAATGGCG
	NGA111 R	CTCCAGTTGGAAGCTAAAGGG
chr.2	PLS5 F	GATGCCTTTCTCCTGGTTG
	PLS5 R	AATATAGCCGTCGTCTTCATCA
	NGA361 F	ACATATCAATATATTAAAGTAGC
	NGA361 R	AAAGAGATGAGAATTTGGAC
	C033 F	CTTATGATAGGGTTTGGTTCC
	C033 R	TGCAATAGCGATATTGGATCG
chr.3	CHIB F	ATGAGAAGCTATAATTTTTTCAATA
	CHIB R	CTCATATATACAAAGAACTACTATAC
	F24M12-TGF F	GTTCTCTGCATTCCACACATACTCT
	F24M12-TGF R	CTTGGGTATTCTGAAGAGCATAAAT
	NGA6 F	ATGGAGAAGCTTACACTGATC
	NGA6 R	TGGATTTCTTCCTCTCTTCAC
	At3g_20318467 F	TGGTAAAGTCAAAGGAAAAGGACG
	At3g_20318467 R	GGATTTGAGCGTTGATGGTAACGAG
	At3g_20424280 F	AAGGTATTGTCCACTCTTGTTCGTG
	At3g_20424280 R	AATGGGTTGGAAATTATGCATC
	At3g_20540707 F	CAGCAAATCCAATAAGTCCATT
	At3g_20540707 R	GGTCCAACAAGTTAGTAACAGAAGT
	At3g_20575005 F	CACATAACTCCCCAGCACAGTAAA
	At3g_20575005 R	CGGAAAGAATTTCAGGGTCTAA
	At3g_20649953 F	CTCCAAGACAAGATCTTCCGTTCT
	At3g_20649953 R	GCTGAAATGGAAGTTCCACAATCA
chr.4	JV30/31 F	CATTAAAATCACCGCCAAAAA
	JV30/31 R	TTTTGTTACATCGAACCACACA
	CIW6 F	CTCGTAGTGCACTTTCATCA
	CIW6 R	CACATGGTTAGGGAAACAATA
	NGA1139 F	TTTTTCCTTGTGTTGCATTCC
	NGA1139 R	TAGCCGGATGAGTTGGTACC
chr.5	NGA106 F	TGCCCCATTTTGTTCTTCTC
	NGA106 R	GTTATGGAGTTTCTAGGGCACG
	SO191 F	CTCCACCAATCATGCAAATG
	SO191 R	TGATGTTGATGGAGATGGTCA
	JV75/76 F	CACAATCAGAGGGGGTTGAT
	JV75/76 R	AAATTTTGGGGGAAATGAAA

Supplemental Table S2. Primers for cloning and sequencing of MKP1

Primer name	Sequence (5' → 3')	Employment
pBIG2113 MKP1 F	GCAGGTCGACTCTAGCGACAAGAAGAGTTTATTCAA	complementation test
pBIG2113 MKP1 R	CCGGGGATCCTCTAGCAGAAACCCTAGAGTAGATC	
MKP1 F	CAATTTCGTGCAGCTAATCAGGAGA	Sequencing of MKP1
MKP1 seq1	GGGAGCTTGATGGTTATTGTTAGA	
MKP1 seq2	GCGAAAGACAAGAGCATACTGA	
MKP1 seq3	GATATGAGAAAGTCGAAGCACCTA	
MKP1 seq4	CATAGTCTTCAATCTGGAGGGTA	
MKP1 seq5	GGACGGTTAGGTAGAATCCTTAT	
MKP1 R	GATTACAAGCCCAGTTGGTGTAGAA	

Supplemental Table S3. Primer sets for qRT-PCR

Primer name	AGI code	Sequence (5' → 3')
Actin2 qRT F	AT3g18780	TCCCTCAGCACATTCCAGCAGAT
Actin2 qRT R		AACGATTCCTGGACCTGCCTCATC
RAB18 qRT F	AT5G66400	CCGTTAAGCTTCGAACAATCGTGT
RAB18 qRT R		CAACACACATCGCAGGACGTACA
RD29A qRT F	AT5G52310	TGGATCTGAAGAACGAATCTGATATC
RD29A qRT R		GGTCTTCCCTTCGCCAGAA
KIN2 qRT F	AT2G02800	TATATCGGATGCGGCAGTGG
KIN2 qRT F		ACAACAAGTACGATGAGTACGAGA
COR15A qRT F	AT2G42450	AGCTTCGGCGCTGTCAGAG
COR15A qRT R		GTCACCTTTAGCGGCGTAGA
CAT1 qRT F	AT1G20630	GACCTCGAGTTCCGACAGTCAA
CAT1 qRT R		CGCCGATTTGCGAGATACACAC
CAT2 qRT F	AT4G35090	CTATCCGACCCACGCATCAC
CAT2 qRT R		TTCAGACGGCTTGCCAGC
APX2 qRT F	AT3G09640	TGGTCGGATGGGACTCAAT
APX2 qRT R		AAGAGCCTTGTCGGTTGGT
GPX7 qRT F	AT4g31870	TCGGCCCATCATTGAGATTC
GPX7 qRT R		CTGCAGCCCTTGCATAGAC
