# Supplementary material for: MAP KINASE PHOSPHATASE1 promotes osmotolerance by suppressing PHYTOALEXIN DEFICIENT4-independent immunity
Source: Plant Physiol. 2022 Mar 18;189(2):1128–38. doi: 10.1093/plphys/kiac131 (PMC9157078; doi:10.1093/plphys/kiac131)
Supplement: kiac131_Supplementary_Data [file kiac131_supplementary_data.zip › Supplementary Figures 13.pdf]

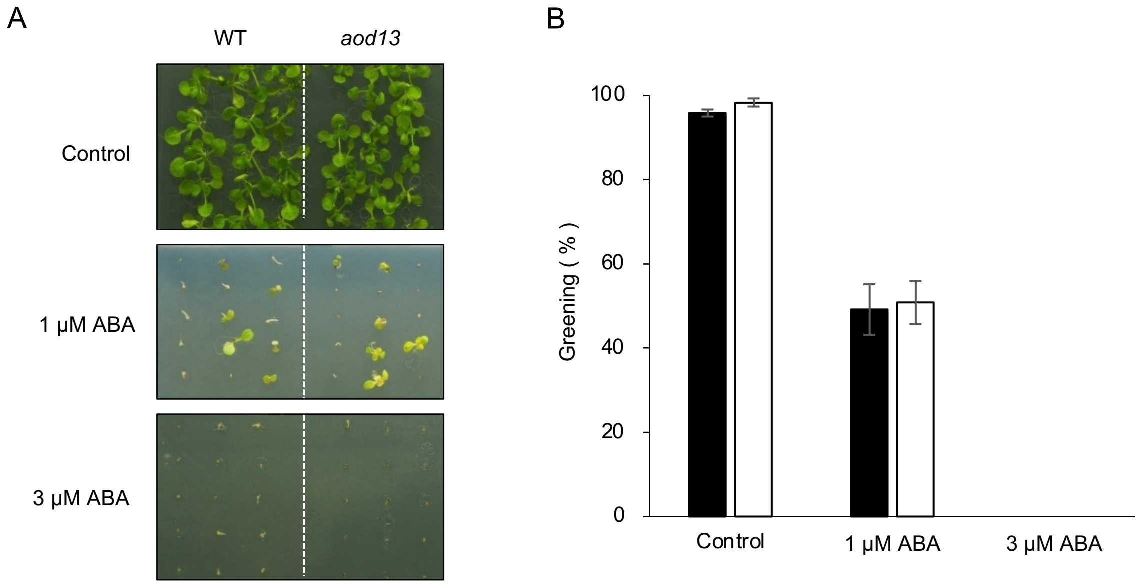

Supplemental Figure S1. Abscisic acid (ABA) sensitivity of the acquired osmotolerance–defective mutant, *aod13*. (A) Two-week-old seedlings of Bu-5 wild type (WT) and *aod13* grown on Murashige and Skoog (MS)–medium agar plates without abscisic acid (ABA) (control) or with 1 or 3 μM ABA. (B) Greening rate (proportion of seedlings that were green) of WT and *aod13* seedlings treated as described in (A). Differences between WT (black bars) and *aod13* (white bars) were analyzed by Student’s t-test (mean ± SE, n = 4).

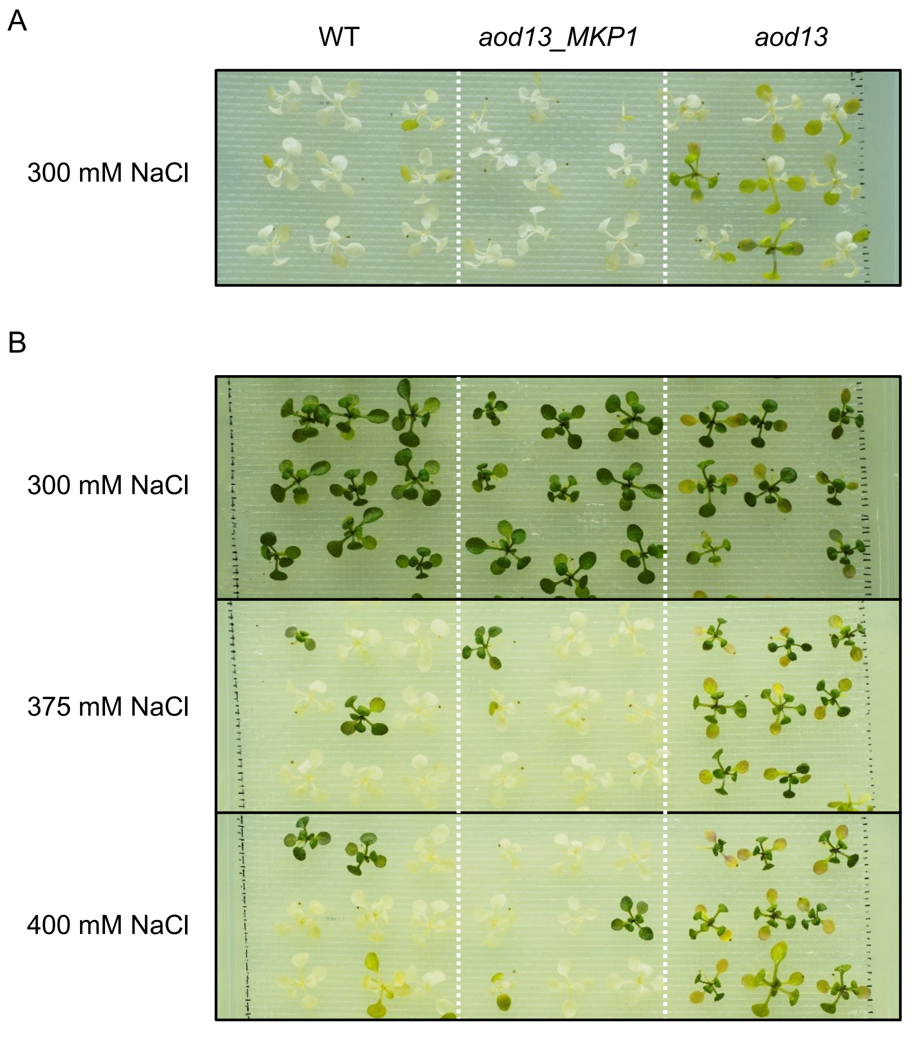

Supplemental Figure S2. Tolerance of *aod13* to severe salt stress. (A) Salt-shock tolerance. Ten-day-old WT, *aod13_MKP1*, and *aod13* seedlings were mesh-transferred to MS agar plates containing 300 mM NaCl for 7 d. (B) Acquired salt tolerance. Seven-day-old seedlings were pre-exposed to 100 mM NaCl for 7 d (acclimation period) and subsequently mesh-transferred to MS agar plated supplemented with 300, 375, or 400 mM NaCl for 7 d.

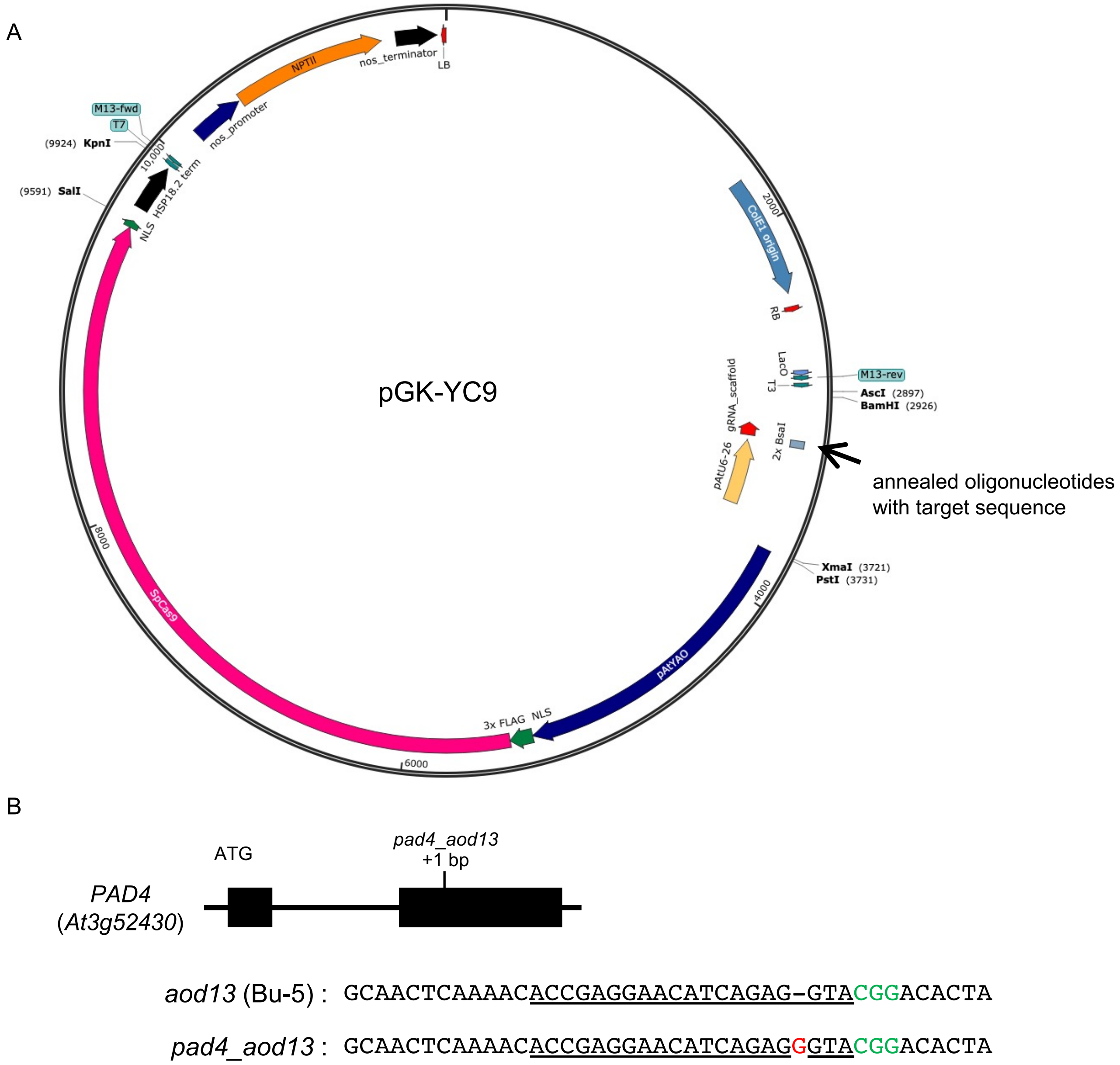

Supplemental Figure S3. Editing of the *PAD4* gene in *aod13* using the CRISPR/Cas9 system. (A) Schematic diagram of pGK-YC9. To achieve high *Cas9* expression in embryos, the *Cas9* gene was driven by the promoter region of the Arabidopsis YAO gene (Yan et al., 2015). The promoter region PCR-amplified from Arabidopsis Col-0 genomic DNA and a codon-optimized SpCas9 with nuclear localization signal sequences cloned from pDe-Cas9 (Fauser et al., 2014) were joined using overlap-PCR. The terminator region of the *AtHSP18.2* gene was cloned from pRI-201AN (Takara Bio). To express single guide RNA (sgRNA) from the *AtU6-26* promoter, an sgRNA cassette was cloned from pUC-AtU6-sgRNA (Endo et al., 2019) with the two *Bbs*I sites replaced by two *Bsa*I sites. These fragments were ligated into the binary vector pGreen0029 (Hellens et al., 2002). The DNA sequence of pGK-YC9 has been deposited in the DNA Data Bank of Japan (DDBJ) with the accession number LC636137. (B) Genomic sequences around the sgRNA target sites of *PAD4*. Schematic diagram (upper panel) and alignment of *PAD4* genes. Black boxes show exons. Indels are indicated as dashes. Red letter shows insertion. Green letters show protospacer adjacent motif (PAM) sequences.
